# Phylogenetic Relationships and Adaptation in Deep-Sea Mussels: Insights from Mitochondrial Genomes

**DOI:** 10.3390/ijms22041900

**Published:** 2021-02-14

**Authors:** Kai Zhang, Jin Sun, Ting Xu, Jian-Wen Qiu, Pei-Yuan Qian

**Affiliations:** 1 Department of Ocean Science, Hong Kong Branch of the Southern Marine Science and Engineering Guangdong Laboratory (Guangzhou), The Hong Kong University of Science and Technology, Hong Kong 93117, China; kayzhang@ust.hk (K.Z.); sunj@ust.hk (J.S.); xuting0708@gmail.com (T.X.); 2 Southern Marine Science and Engineering Guangdong Laboratory (Guangzhou), Guangzhou 510225, China; 3 Department of Biology, Hong Kong Baptist University, Hong Kong 93117, China

**Keywords:** adaptation, deep-sea, extreme environment, mitochondrial genome, mussel

## Abstract

Mitochondrial genomes (mitogenomes) are an excellent source of information for phylogenetic and evolutionary studies, but their application in marine invertebrates is limited. In the present study, we utilized mitogenomes to elucidate the phylogeny and environmental adaptation in deep-sea mussels (Mytilidae: Bathymodiolinae). We sequenced and assembled seven bathymodioline mitogenomes. A phylogenetic analysis integrating the seven newly assembled and six previously reported bathymodioline mitogenomes revealed that these bathymodiolines are divided into three well-supported clades represented by five *Gigantidas* species, six *Bathymodiolus* species, and two “*Bathymodiolus*” species, respectively. A Common interval Rearrangement Explorer (CREx) analysis revealed a gene order rearrangement in bathymodiolines that is distinct from that in other shallow-water mytilids. The CREx analysis also suggested that reversal, transposition, and tandem duplications with subsequent random gene loss (TDRL) may have been responsible for the evolution of mitochondrial gene orders in bathymodiolines. Moreover, a comparison of the mitogenomes of shallow-water and deep-sea mussels revealed that the latter lineage has experienced relaxed purifying selection, but 16 residues of the *atp6*, *nad4*, *nad2*, *cob*, *nad5*, and *cox2* genes have underwent positive selection. Overall, this study provides new insights into the phylogenetic relationships and mitogenomic adaptations of deep-sea mussels

## 1. Introduction

Mytilidae is a family of highly diverse mussels that are widely distributed from freshwater to marine water and from coastal areas to the deep-sea [1,2]. This family is currently divided into seven subfamilies. Although most of these subfamilies are represented by shallow-water species, the subfamily Bathymodiolinae consists of exclusively deep-sea species [2,3]. A previous study has established that Bathymodiolinae split from its shallow-water sister group Modiolinae roughly 110.4 Million years ago (Ma) [4], but given the difficulty in sampling these deep-sea animals, little is known about their biology. Members of Bathymodiolinae, especially those belonging to the genera *Bathymodiolus, Gigantidas, Idas*, and *Tamu*, are often conspicuous animals in chemosynthesis-based ecosystems, including hydrothermal vents, cold seeps, and organic falls [5,6]. Despite the lack of phytoplankton-derived food in the deep-sea, these mussels thrive in the special deep-sea habitats due to their symbiosis with bacteria that are dependent on simple organic molecules, such as methane and hydrogen sulfide, as a source of energy [1,4]. Owing to their ecological importance and remarkable biological characteristics, deep-sea mussels have been considered a suitable animal model for studying adaptation and symbiosis [4,7,8,9,10].

The taxonomy of Bathymodiolinae is in disarray because of their high morphological plasticity. Molecular phylogenetic studies have divided deep-sea mussels previously referred to as *Bathymodiolus* into nine genera, namely, *Gigantidas*, *Bathymodiolus*, *Adipicola*, *Benthomodiolus*, *Idas*, *Tamu*, *Terua*, *Vulcanidas*, and “*Bathymodiolus*” [5,11,12]. “*Bathymodiolus*”, which is currently represented by only two species (“*B.*” *aduloides* and “*B.*” *manusensis*), is vastly different from *Bathymodiolus* sensu stricto, but it has not been given a formal genus name yet [6,12,13].

Previous phylogenetic studies of deep-sea mussels mainly used one or very few gene fragments. Consequently, they provided limited information on the evolutionary history of these animals. In the present study, we examined the phylogenetics and evolution of the deep-sea mussels based on mitochondrial genomes (mitogenomes). Mitogenomes carry useful evolutionary information and have been widely applied in phylogenetic and evolutionary studies of terrestrial and shallow-water organisms, including mussels [14,15,16,17]. Given that the mitochondria play a key role in the energetic metabolism of metazoans, the hypoxia and high hydrostatic pressure environments in cold seeps and hydrothermal vents could exert selective pressure on the evolution of these energy-producing organelles [18,19,20,21]. However, only six Bathymodiolinae mitogenomes are currently available, thereby hindering our understanding of their evolution. 

In the present study, we sequenced seven complete Bathymodiolinae mitogenomes and analyzed them together with the previously published mitogenomes to infer their phylogenetic relationships, understand their gene order rearrangement patterns, determine their divergence times, and ascertain the adaptive evolution of protein-coding genes (PCGs).

## 2. Results

### 2.1. Genome Organizations and Gene Rearrangement

**Figure 1:**
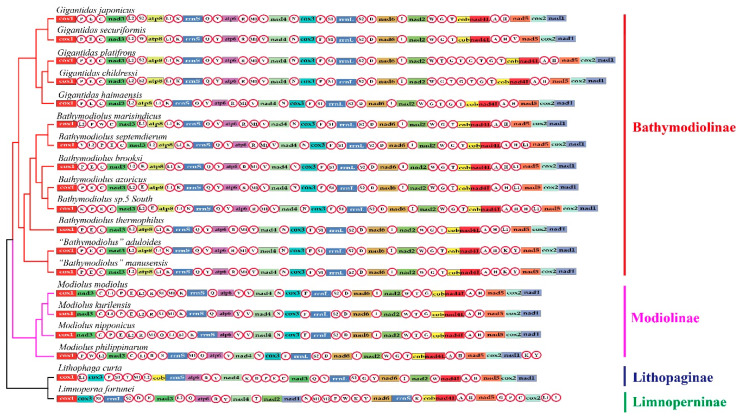
Linearized mitochondrial gene arrangement patterns of the investigated species superimposed on the phylogenetic tree. Gene and genome size are not in scale. The tRNAs are labeled by single-letter abbreviations of the amino acid code.

The size of the seven newly assembled mitogenomes ranged from 17,138 bp (*B. marisindicus*) to 18,376 bp (*B.* sp. 5 South) (Table 1). They all included 13 PCGs and two rRNA genes. However, the number of tRNA genes varied (23–28), an observation consistent with that reported for other groups of bivalves [22,23]. Within the *Gigantidas* group, some species had multiple copies of *trnT* and *trnG*: *G. platifrons* had three copies of *trnG* and four copies of *trnT*, *G. childressi* had three copies of *trnG* and *trnT*, and *G. haimaensis* had two copies of *trnG* and *trnT*. These copies of *trnT* and *trnG* were located between *trnW* and *cob*. Within the *Bathymodiolus* group, all species had two copies of *trnL1*, except for *B. marisindicus.* Both species of the “*Bathymodiolus”* group had two copies of *trnK* and *trnY*. A comparison among the bathymodioline mitogenomes revealed that they had a highly conserved gene order, except for the translocation or inversion of some tRNAs (Figure 1). The four *Modiolus* species and *L. curta* from shallow seawater and *L. fortunei* from freshwater lacked the *atp8* gene (Figure 1). Regions homologous to the *atp8* gene were detected in the mitogenomes of four *Modiolus* species using the Align by Muscle model implemented in MEGA v.7.0. These regions were located between *nad1* and *cox1*, but the gene structure was incomplete.

**Figure 2:**
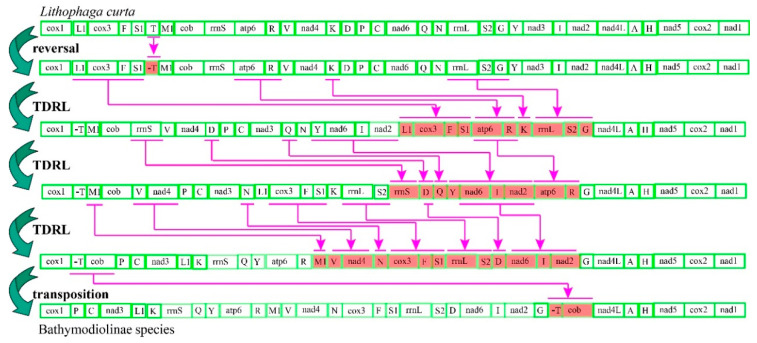
Putative gene rearrangement events from *Lithophaga curta* to Bathymodiolinae species. Purple lines and genes are colored to indicate tandem duplication random loss (TDRL) events step by step as identified by Common interval Rearrangement Explorer (CREx) analysis.

Common interval Rearrangement Explorer (CREx) analysis was performed to determine the likely gene order rearrangement events that occurred during the evolution of deep-sea mussels. After incomplete and duplicated gene constitutions were removed, six unique gene arrangements among the 19 mitogenomes analyzed were detected. All bathymodioline mitogenomes possessed a gene order notably different from that of other mussels. Three gene order rearrangements were detected in the four *Modiolus* species, indicating that this group has a higher diversity of gene orders than the other groups. We designated the gene order of *L. curta* as the ancestral Bathymodiolinae gene order for CREx analysis because it is an outgroup taxon that shares a similar gene order with Bathymodiolinae and Modiolinae species [2]. CREx analysis suggested that the distinct gene order of bathymodioline mussels might have evolved from the putative ancestral bathymodioline gene order through five evolutionary steps (Figure 2). These steps included one reversal, one transposition, and three complex tandem duplications with subsequent random gene losses (TDRLs). Aside from the recurrent rearrangements of tRNAs, rearrangements were also observed in the PCGs. Specifically, the gene cluster *cox3+trnF+trnS1* moved from between *trnL1* and *trnT* to a position between *trnN* and *rrnL*, *nad3* moved from between *trnY* and *trnI* to a position between *trnC* and *trnL1*, and *cob* moved from between *trnM1* and *rrnS* to between *trnT* and *nad4L*. When the tRNA genes were excluded from the comparisons, three conserved gene blocks (A, *trnV*–*nad4*–*trnN*–*cox3*–*trnF*; B, *rrnL*–*trnS2*–*trnD*–*nad6-I*–*nad2*; C, *cob*–*nad4L*–*trnA*–*trnH*–*nad5*–*cox2*–*nad1*) were identified in the mitogenomes of Bathymodiolinae and Modiolinae species.

### 2.2. Phylogenetic Relationships and Divergence Times

A total of 3243 amino acid positions were found in the aligned sequence dataset. The best sequence evolution models identified herein by using PartitionFinder included JTT+G+F for *atp6*, *nad2*, *nad3*, *nad4L*, *nad5*, and *nad6*; LG+G+F for *cox2, cox3*, and *nad1*; and MTART+G+F for *cob*, *cox1*, and *nad4* (Table S1).

**Figure 3:**
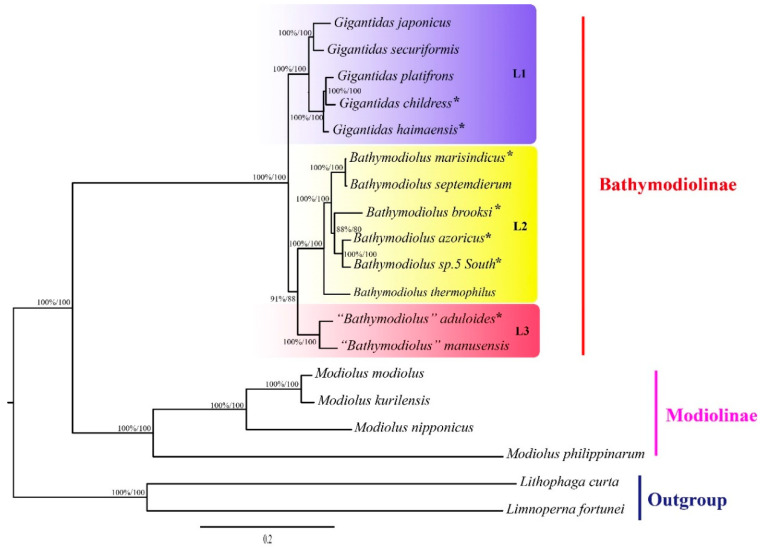
Phylogenetic tree of the deep-sea and shallow-water mussels inferred from 12 protein genes by maximum likelihood. Bayesian posterior probabilities/maximum likelihood (ML) bootstrap support values are shown for each node. An asterisk denotes mtDNA sequence newly determined in this study. Scale bar represents nucleotide substitutions per site.

**Figure 4:**
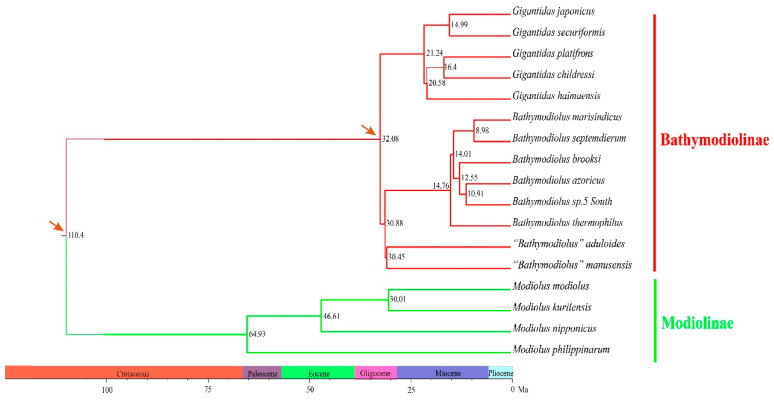
Molecular dating of species divergence events within the Bathymodiolinae and Modiolinae. Calibration points are marked by an arrow.

The phylogenetic trees constructed using both the maximum likelihood (ML) and Bayesian inference (BI) methods consistently showed that bathymodioline mussels analyzed were divided into three separate clades (L1, L2, and L3) with high support values (Figure 3). Molecular dating results indicated that the three clades diversified in the last 32.08 Ma (Figure 4). The five *Gigantidas* species formed the L1 clade, which included species from cold seeps and those that harbor mainly methane-oxidizing symbionts. The *Gigantidas* clade diverged from the other bathymodiolines, approximately 21.24 Ma. The L2 clade consisted of six *Bathymodiolus* species, including *B. marisindicus*, *B. septemdierum*, and *B. azoricus*, as well as a species from hydrothermal vents that has not been formally described (*Bathymodiolus* sp. 5 South), and *B. brooksi* from cold seeps. Two species from hydrothermal vents, namely, “*Bathymodiolus” aduloides,* and “*Bathymodiolus” manusensis*, comprised the L3 clade. These results are consistent with those of a previous study that showed that these two species do not belong to *Bathymodiolus* [6] but had higher bootstrap values in the tree. The present estimate for the divergence between “*Bathymodiolus*” mussels and *Bathymodiolus* mussels was 30.88 Ma (Figure 4). 

### 2.3. Genetic Distance

The Kimura-2-parameter (K2P) distance of the *cox1* sequences of the bathymodiolines analyzed herein varied from 0.8% to 18.85%, whereas that for *Modiolus* species ranged from 12.33% to 33.36% (Table S2). The K2P distance of the combined PCG sequences varied from 1.04% to 23.16% for the bathymodiolines and from 11.39% to 44.82% for the modiolines. Among the bathymodiolines, the smallest genetic distance was between *B. marisindicus* and *B. septemdierum* regardless if it was based on *cox1* or the combined PCG sequences. Based on the *cox1* gene, the highest genetic distances among the three major bathymodioline clades were 17.99% (between the L1 and L2 clades), 16.58% (between the L1 and L3 clades), and 18.85% (between the L2 and L3 clades).

### 2.4. Elevated Nucleotide Substitution Rates in Deep-Sea Mussels

CodeML from the PAML package was used to evaluate whether positive selection might have contributed to the adaptation of mussels to deep-sea environments. No significant differences were observed between the one- and two-ratio models of ω (*Ka/Ks*) for all PCGs (Table S3). This result suggested that ω did not change more quickly than expected along the Bathymodiolinae branch. Moreover, the ω values of all mitochondrial PCGs in both models were substantially lower than 1, ranging from 0.00105 to 0.14038 for all PCGs for the deep-sea bathymodiolines and from 0.00239 to 0.06698 for the shallow-water mussels. Nevertheless, compared with shallow-water mussels, the deep-sea mussels had higher ω values for all PCGs, except for *cox1*.

Branch-site models were employed to detect positively selected sites in the mitogenomes of deep-sea mussels. Sixteen residues located on *atp6*, *nad4*, *nad2*, *cob*, *nad5*, and *cox2* were detected as positively selected sites along the Bathymodiolinae branch (>95%) (Table 2**)**. These results suggested that the bathymodiolines were affected more heavily than the shallow-water species after they diverged from their common ancestor.

## 3. Discussion

### 3.1. General Features of Bathymodiolinae Mitogenomes

Seven complete Bathymodiolinae mitogenomes ranging in length from 17,138 bp (*B. marisindicus*) to 18,376 bp (*B.* sp. 5 South) were newly assembled in the present study. This narrow range of genome size was consistent with that of a previous study that reported that the size of the mitogenomes of four other bathymodioline species ranging from 17,069 bp to 18,819 bp [2,24]. The variations in genome size were mainly attributed to the size differences in the control region [2], ranging from 469 bp in *B. marisindicus* to 1963 bp in *G. haimaensis*. The arrangement and number of tRNA genes are highly variable among different mussel subfamilies, which is also true for other bivalves [2,22]. Nevertheless, when tRNA genes are not included, the mitogenomes of mussels in Bathymodiolinae and Modiolinae have similar gene contents and gene order arrangements. However, although *atp8* is present in most of the sequenced mitogenomes of mussels in other subfamilies of Mytilidae, including all species of deep-sea mussels, this gene is missing in some species of Modiolinae, Lithophaginae, Limnopernimae, and Brachidontinae [2], or has become a pseudogene [25].

### 3.2. Molecular Phylogeny of Deep-Sea Mussels

The topologies inferred from the amino acid sequences of PCGs via the BI and ML methods were consistent. Results showed that the deep-sea mussels investigated could be divided into three clades (L1, L2, and L3) with high branch support values (Figure 3). These results are consistent with the findings of earlier studies [5,12], which support the classification of deep-sea mussels that species in the L3 clade belong to *Nipponiomodiolus* and those in the L1 clade belong to *Gigantidas* [6,12,13]. Nevertheless, the present results do not completely concur with those of previous studies that analyzed a combined dataset of mitochondrial and nuclear gene sequences [3,6]. For instance, the present study placed *Gigantidas* (L1 clade) as the sister group of (L2 + L3); in contrast, a recent molecular analysis using a combined dataset of mitochondrial and nuclear gene sequences indicates that this genus is closely clustered with the L2 clade [3]. Moreover, *B. aduloides* and *B. manusensis* (L3 clade) form a sister group to the L2 clade. This result is consistent with that of our previous works [12,13] but in stark contrast to that of some earlier phylogenetic analyses of few mitochondrial and nuclear datasets that argued that these two species are more closely related to the L1 clade (*Gigantidas*) [5,26]. Furthermore, the tree topology test rejected the hypothesis that the L3 clade is sister to the L1 clade (Table S4). 

CREx analysis revealed that all bathymodioline species analyzed herein had identical gene order arrangements, a remarkable result because the gene orders in many other groups of Mytilidae substantially vary [2]. These deep-sea bathymodiolines are phylogenetically closely related, indicating that their gene orders have not changed since their common ancestor diverged from other lineages of mussels. The K2P genetic distance between the bathymodiolines analyzed herein varied from 0.8% to 18.95% for *cox1* and from 1.04% to 23.16% for all PCGs. These results demonstrated that these bathymodiolines (L1 + L2 + L3) are a monophyletic group, consistent with the findings of previous studies [3,13,26]. Unlike Bathymodionae species, which have only one gene order, the Modiolinae species analyzed herein have three gene orders, and their K2P genetic distances varied from 11.39% to 44.82% for PCGs. Nevertheless, the taxonomic distribution of the available samples in this study was biased toward Bathymodiolinae, and several genera of this subfamily were not included in the analysis due to the lack of access to specimens. 

DNA barcoding is widely used for species delimitation [27]. Thubaut et al. (2013) used a 2.0% K2P genetic distance as the threshold for species delimitation in bathymodioline mussels [6]. However, among the recognized species, the genetic divergence between *B. septemdierum* and *B. marisindicus* is 0.8% for the *cox1* gene and 1.04% for all PCGs (Table S2). Therefore, these two species are likely conspecific [26,28]. 

### 3.3. Mitochondrial Gene Rearrangement

Mollusks have been utilized as animal models for investigating mitogenome gene order rearrangements [29,30]. In the present study, a novel gene order was observed in the mitogenomes of deep-sea Bathymodiolinea mussels that greatly differed from the gene orders of other mussel groups. Although these deep-sea mussels have an identical mitochondrial gene order, the shallow-water mussels used in this study possess three mitochondrial gene orders, suggesting only one rearrangement following the divergence of bathymodiolines from the other mussels. Given that the mitochondrial gene order in deep-sea Bathymodiolinae species is different from that of shallow-water modioline mussels, including *Modiolus* and *Lithophaga*, we speculate that this unique gene order rearrangement pattern might have occurred after the shallow-water mussels invaded the deep-sea. Future studies should examine the mitochondrial gene order of other genera of deep-sea mussels, especially *Benthomodiolus* species, which usually inhabit sunken woods or whale falls that are considered as transitional habits to the most specialized vent and seep habitats [5,6]. 

Mitochondrial gene order arrangement involves four gene rearrangement types, namely, transpositions, reverse transpositions, inversions, and TDRL [31,32]. CREx analysis suggested that transposition and TDRL are associated with the evolution of the mitogenomes of deep-sea mussels. These substantial rearrangements indicated that dramatic mitogenome organizations occurred during the invasion of the deep-sea by mussels. This result is consistent with that of other studies of deep-sea species showing their gene orders were also altered, including tRNA and PCG transportation or gene cluster invasion, during their invasion into the deep-sea [33,34,35]. How such gene order rearrangements might be adaptive to deep-sea mussels is unknown. Nevertheless, gene recombination has been suggested to enhance the survival of deep-sea species by offsetting the high mutational rates of mitochondrial DNA [17,36].

### 3.4. Adaptations to Deep-Sea Environments

As reported by earlier studies, mitochondrial PCGs may experience positive selection in deep-sea animals and thus may help them adjust their metabolism to tolerate the deep-sea conditions [37,38,39]. In the present study, potential positive selection was evaluated in deep-sea mussels by using CodeML in the PAML package. Analyses of branch models showed that the *Ka/Ks* ratios for all PCGs in both one- and two-ratio models were substantially lower than 1 (Table S3), implying strong purifying selection has driven the evolution of the mitogenomes of these mussels. In addition, the ω values for all PCGs, except for the *cox1* gene, in deep-sea mussels were higher than those in sublittoral species, indicating that the mitogenomes of deep-sea mussels underwent a more relaxed purifying selection. Low ω values for mitochondrial PCGs were also reported in other deep-sea animals, including deep-sea vesicomyids [3,21], a giant *Bathynomus* sp. [40], and deep-sea polynoids [35]. Moreover, previous studies found that positive selection usually occurs within a short period of evolutionary time and acts on only a few sites. Thus, the sparse signals of positive selection are usually overwhelmed by those for continuous purifying selection on most sites in a gene sequence [41,42].

Branch-site models are used to identify possible positively selected sites in deep-sea mussels. Results suggested that 16 residues located in the *atp6*, *nad4*, *nad2*, *cob*, *nad5*, and *cox2* genes could have experienced positive selection along the branch ancestral to Bathymodiolinae. As a proton pump, the NADH dehydrogenase complex is the largest and foremost enzyme complex of the respiratory chain. The efficiency of proton pumping procedures can be affected by protein mutation, and thus it may be crucial to adaptive evolution [43,44]. In the deep-sea shrimp family Alvinocarididae, the greatest residues of positively selected sites are within *nad1-5* [39], a gene also known to be related to deep-sea hydrothermal vent adaptation. The *nad3* and *nad5* genes in the mitogenome of the deep-sea crab *Chaceon granulates* also harbor positively selected residues [20]. Similarly, 11 residues are considered positively selected in the *nad2* and *nad4* genes of the deep-sea sea cucumber *Benthodytes marianensis* [39]. Cytochrome c oxidase stimulating the terminal reduction of oxygen and with three mitochondrial PCGs (*cox1*, *cox2*, and *cox3*) that encode the catalytic core is an important positive selection target in hypoxia adaptation [45,46]. In deep-sea clams belonging to the family Vesicomyidae, positively selected residues are found in the *cox1* and *cox3* genes [20]. The ATP synthase F_o_ subunit 6 or complex V drives the last step of oxidative phosphorylation for electron transport chain. Evidence supporting the adaptive evolution of the *atp6* gene has been reported in the mitogenome of *Glyptothorax macromaculatus* [47], deep-sea fish [19], and deep-sea polynoids [20]. Although *cob* is a conserved gene, it is crucial to the ability of the mitochondria to generate energy through reversible electron transfer from ubiquinol to cytochrome c along with proton translocation. *cob* was shown to have undergone positive selection in deep-sea fish [19] and deep-sea clams [21]. Therefore, mitochondrial genes, particularly *atp6*, *nad4*, *nad2*, *cob*, *nad5*, and *cox2*, may help deep-sea mussels to survive and/or thrive under harsh deep-sea conditions.

## 4. Materials and Methods

### 4.1. Acquisition of Mitochondrial Genome Sequences

We analyzed the mitogenomes of 19 species of mussels, 13 of which were deep-sea bathymodiolines (Table 1). Six of the mitogenomes were downloaded from GenBank, whereas seven were newly assembled herein. Out of the newly assembled mitogenomes, four were based on DNA sequences downloaded from GenBank (*Bathymodiolus* sp. 5 South (ERP115508), *B. azoricus* (ERP105025), *B. brooksi* (SRP178172), *G. childressi* (ERP021949)), and three were based on DNA sequences produced herein: “*Bathymodiolus” aduloides* was collected from the F-site methane seep in the South China Sea (22°06.921′ N, 119°17.131′ E, depth 1122 m), *B. marisindicus* was gathered from the Longqi hydrothermal vent field in the Southwest Indian Ocean Ridge (37°47′ S 49°39′ E, depth 2800 m), and *G. haimaensis* was obtained from the Haima Cold Seep in the South China Sea (16°44′ N, 110°29′ E, depth 1390 m). The adductor muscle of an individual of these three species was dissected, and its DNA was extracted using the DNeasy Blood and Tissue Kit (Qiagen, Hilden, Germany). DNA library construction and sequencing were conducted by Novogene, Beijing. Sequencing was conducted in paired-end mode on an Illumina platform to produce approximately 5 Gb of reads of 150 bp read length. Whole genome sequencing datasets were assembled using NOVOPlasty v.3.8.3 under default settings [48]. 

### 4.2. Genome Sequence Annotation and Gene Arrangement Analysis

The MITOS webserver was used to annotate the mitogenomes assembled herein [49]. The boundaries of 13 PCGs and two ribosomal RNA (rRNA) genes were determined by comparing them with the homologous genes of other bathymodioline species. Transfer RNA (tRNA) genes were predicted using the programs MiTFi [50] in the MITOS pipeline [49], ARWEN v1.2.3.c [51], and tRNAscan-SE v1.21 [52]. The gene order of *Lithophaga curta* mitogenome was considered as the ancestral gene order of Bathymodiolinae. Pairwise comparisons of mitogenomes were conducted using Common interval Rearrangement Explorer (CREx) [53] to reconstruct the likely gene order rearrangement events (i.e., reversal, transposition, reverse transposition, tandem duplication/random loss (TDRL)) that might have occurred during the evolution of this lineage of mussels.

### 4.3. Phylogenetic Analyses and Divergence Time Estimation

Out of the 19 mitochondrial genome sequences used for phylogenetic analyses, two were outgroups (*L. curta* and *Limnoperna fortunei*). The amino acid sequences of 12 PCGs were allied separately by using MAFFT v.7.407 [54] under defaults settings. The *atp8* gene was not included in the analysis because shallow-water mussels lack this gene. Poorly aligned positions were removed using Gblocks v.0.91b [55]. The best-fit substitution models for the dataset and the best partition schemes were determined using PartitionFinder version 2.1.1 (Australian National University, Canberra, ACT, Australia) [56]. 

Phylogenetic relationships were reconstructed via the Maximum Likelihood (ML) method implemented in RAxML v.8.2.9 [57] and the Bayesian inference (BI) method implemented in MrBayes v.3.2.7a [58]. For the ML analysis, 10,000 replicates were employed. For the BI analysis, the Markov chain Monte Carlo (MCMC) method was applied considering a chain for 10 million generations, and a tree was sampled every 500 generations. The initial 25% of the runs were discarded as burn-in. Alternative tree topologies were assessed via the approximately unbiased test implemented in IQ-TREE v.2.0 [59] with 20,000 bootstrap replicates. After phylogenetic tree construction, the timing of species divergences was estimated via the Bayesian method by using MCMCTree in the PAML package v.4.9h [60]. Two nodes were time-calibrated. According to previous studies [4,7], bathymodioline and shallow-water mussels split about 110.4 Ma, and *G. childressi* and *B. thermophilus* diverged between 21.12 and 32.98 Ma. For the MCMC analysis, 100,000 samples were applied, and the first 20% of all samples were discarded as burn-in. An independent rate model (clock = 2), which follows a lognormal distribution, was used for the MCMC search. The phylogenetic tree was visualized in FigTree v.1.4.3 [61].

Genetic distances between the species tested herein were computed using the Kimura-2-parameter (K2P) model (Kimura, 1980) implemented in MEGA v.7.0 [62] for both *cox1* and the combined mitochondrial PCGs.

### 4.4. Positive Selection Analysis

Positive selection in the branches leading to the deep-sea Bathymodiolinae was determined using the branch model and the branch-site model in the PAML package [60]. Selection pressure was determined by applying the overall database of 12 mitochondrial PCGs. The topology of the phylogenetic tree generated in the previous section was utilized in this analysis. For the branch model, the one-ratio model (model = 0, assuming a single ω0 ratio for all branches in the phylogenetic tree) was used to assess ω distribution values (*dN/dS* ratio), which were taken as the basis for the probability of adaptive evolution of gene sequences. Subsequently, the two-ratio model (model = 2, setting the bathymodiolinae branch as foreground lineages, ω2; setting all other branches as background lineages, ω1) was ran. Furthermore, the one- and two-ratio models were compared to investigate whether the clade of deep-sea mussels is under greater selection pressure than shallow-water mussels. If the two-ratio model showed a significantly higher probability than the one-ratio model and ω2 > 1, then the deep-sea mussels were considered to be under positive selection. Afterward, positively selected sites in the deep-sea Bothymodiolinae lineage (marked as foreground lineage) were determined via a branch-site model. Bayesian posterior probability of the positively selected sites was obtained via Bayes Empirical Bayes (BEB) analysis.

## 5. Conclusions

In summary, the mitogenomes of various genera of Bathymodiolinae were found to have a conserved gene order, which differs remarkably from the gene orders of shallow-water mussels. Our results suggested that gene order rearrangements in bathymodiolines can be explained by reversal, transposition, and TDRL of an ancestral mitogenome. Finally, multiple mitochondrial genes carry signals of positive selections in some amino acid residues in deep-sea mussels, a condition indicating adaptation to deep-sea environments.

## Figures and Tables

**Table 1:** Complete mitochondrial genomes used for phylogenetic analysis in this study.

Species	Subfamily	bp	Accession No.	Reference
*Gigantidas japonicus*	Bathymodiolinae	17,510	AP014560	Robicheau et al. (2017)
*Gigantidas platifrons*	Bathymodiolinae	17,653	AP014561	Robicheau et al. (2017)
*Bathmodiolus septemdierm*	Bathymodiolinae	17,069	AP014562	Robicheau et al. (2017)
*Bathymodiolus thermophilus*	Bathymodiolinae	18,819	MK721544	Lee et al. (2019)
*Bathymodiolus securiformis*	Bathymodiolinae	17,199	NC_039552	-
*Bathymodiolus manusensis*	Bathymodiolinae	16,801	KY270856	-
*Bathymodiolus* sp. 5 South	Bathymodiolinae	18,376	MT916740	This study
*Bathymodiolus aduloides*	Bathymodiolinae	17,243	MT916741	This study
*Bathymodiolus azoricus*	Bathymodiolinae	17,598	MT916742	This study
*Bathymodiolus brooksi*	Bathymodiolinae	17,728	MT916743	This study
*Bathymodiolus childressi*	Bathymodiolinae	17,637	MT916744	This study
*Bathymodiolus marisindicus*	Bathymodiolinae	17,138	MT916745	This study
*Gigantidas haimaensis*	Bathymodiolinae	18,283	MT916746	This study
*Modiolus modiolus*	Modiolinae	15,816	KX821782	Robicheau et al. (2017)
*Modiolus kurilensis*	Modiolinae	16,210	KY242717	-
*Modiolus nipponicus*	Modiolinae	15,638	MK721547	Lee et al. (2019)
*Modiolus philippinarum*	Modiolinae	16,389	KY705073	Sun et al. (2017)
*Lithophaga curta*	Lithophaginae	16,580	MK721546	Lee et al. (2019)
*Limnoperna fortunei*	Limnoperninae	18,145	KP756905	Uliano-Silva et al. (2016)

**Table 2:** Possible sites under positive selection in the deep-sea mussels.

Gene	Codon	Amino Acid	BEB Values
*atp6*	50	L	0.994
*nad4*	76	P	0.992
	234	K	0.991
*nad2*	34	S	0.991
	37	S	0.995
	59	K	0.991
	60	S	0.994
	122	W	0.997
	331	G	0.992
*cob*	54	S	0.991
	317	N	0.991
	351	L	0.994
*nad5*	85	S	0.995
	439	S	0.997
	503	S	0.991
*cox2*	95	K	0.993

## Data Availability

The sequence data have been deposited in the NCBI database with accession numbers MT916740, MT916741, MT916742, MT916743, MT916744, MT916745 and MT916746.

## Supplementary Materials

The following are available online at https://www.mdpi.com/1422-0067/22/4/1900/s1, Table S1: The best schemes and best-fit substitution model for phylogenetic tree, Table S2: Pairwise genetic distance (%) between species belonging to the genera *Gigantidas*, *Bathymodiolus*, *Modiolus*, *Lithophaga* and *Limnoperna*, Table S3: Models of selection pressure on the mitochondrial protein-coding genes of the deep-sea mussels, Table S4: The result of approximately unbiased (AU) test.
